# Understanding knowledge, beliefs, values and barriers towards cervical cancer screening and self-sampling amongst migrant Muslim women in Southwest London: an in-depth qualitative interview study

**DOI:** 10.1136/bmjph-2025-003254

**Published:** 2026-01-09

**Authors:** Sophie Webb, Nafeesa Mat Ali, Yolanda Augustin, Sally E Hayward, Anna Deal, Alison Crawshaw, Henry Staines, Kevin Hayes, Sally Hargreaves, Sanjeev Krishna

**Affiliations:** 1 Institute of Infection and Immunity, City St George’s University of London, London, England, UK; 2 St George’s University Hospitals NHS Foundation Trust, London, England, UK; 3 City St George’s University of London, London, UK; 4 Institut für Tropenmedizin, Universitätsklinikum Tübingen, Tübingen, Germany; 5 Centre de Recherches Médicales de Lambaréné, Lambarene, Moyen-Ogooue, Gabon

**Keywords:** Human Papillomavirus Viruses, Mass Screening, Sexual Health, Qualitative Research

## Abstract

**Intro:**

Novel screening methods are needed to increase access to cervical screening, and migrant Muslim women in the UK are particularly at risk of screening non-attendance. In anticipation of the introduction of high-risk human papillomavirus (hrHPV) self-sampling into the UK programme, this study explored views of migrant Muslim women in southwest London on understanding of cervical screening, barriers and motivators to engagement and acceptability of vaginal self-sampling.

**Methods:**

Qualitative in-depth semi-structured individual interviews were carried out via MS Teams video call of 18 Muslim migrant women, with purposive and snowball recruitment. Framework analysis was carried out using NVivo 14 and coding matrix developed using MS Excel.

**Results:**

Migrant Muslim women felt that self-sampling for hrHPV was likely acceptable and beneficial for some women in their community. Only 44% preferred self-sampling over healthcare worker (HCW)–taken samples because of concerns over technique and inadequate results. There was a lack of understanding of the screening programme, role of HPV and cultural taboo of sexual activity outside of marriage. These barriers may be mitigated by evidence-based information in their own language by a trusted HCW or community champion. Taking their own respiratory swabs during the COVID-19 pandemic made participants more open-minded to self-sampling.

**Conclusions:**

Low vaginal self-sampling is acceptable to migrant Muslim women; however, over half may still prefer HCW-taken samples. Key strategies for overcoming barriers to self-sampling are prioritising linguistically appropriate materials, partnership with community leaders, flexible access points to screening and confidential modes of result delivery.

**WHAT IS ALREADY KNOWN ON THIS TOPIC**

- No studies in the UK have looked specifically at the views of migrant Muslim women on self-sampling for cervical screening, and they are at increased risk of screening non-attendance and inequitable access to healthcare.

**WHAT THIS STUDY ADDS**

- Migrant Muslim women find low vaginal self-sampling for high-risk human papillomavirus (hrHPV) acceptable and acknowledge that it is likely to be beneficial for members of their community who struggle with traditional healthcare worker (HCW)–taken samples.
- Owing to concerns over technique, inadequate results and lack of understanding of the screening programme, as well as cultural taboo, over half of the women still preferred a HCW taken screening sample to a self-sample.

**HOW THIS STUDY MIGHT AFFECT RESEARCH, PRACTICE OR POLICY**

- The UK National Screening Committee recently recommended offering HPV self-sampling to under-screened people as part of the cervical screening programme.
- This study demonstrates the specific views of migrant Muslim women on self-sampling and gives patient-centred strategies to improve access and uptake of cervical screening, including HPV self-sampling as it is rolled out in the UK.

## Introduction

 Cervical cancer accounts for 2% of all new cancer cases in females in the UK.^1^ The estimated lifetime risk of this diagnosis for women born after 1960 is 1 in 142.^2^ The COVID-19 pandemic resulted in delays to the NHS Cervical Screening Programme (NHSCSP) in the UK, with 4 67 867 women estimated to have had their screening disrupted during the acute phase of the pandemic. The latest projections show that despite recovery strategies prioritising women on early recall pathways, the disruption may result in 4.2 excess cancers per 1 00 000 women who attended the NHSCSP, with a maximum delay to their screening of 12 months in England.^3^

Given that 99.8% of cervical cancer is due to high-risk human papilloma virus (hrHPV) infection,^4^ early detection and treatment of persistent hrHPV infections is crucial to preventing cervical cancer. In December 2019, England moved to hrHPV testing in place of liquid-based cytology screening; however, the screening coverage for all women of screening age (25–64 years) was only 68.8% in 2023–2024.^5^ This is well below the NHSCSP standard of ≥80%.^6^ The UK National Screening Committee has recently recommended offering hrHPV self-sampling to those who delay their attendance by >6 months, or who never attend, as these people are at higher risk of being affected by HPV, though it has allowed each local service to decide whether or not to roll this out to their individual population.^7 8^

Socio-economic deprivation often results in healthcare inequity and is a significant barrier to accessing the NHSCSP. Women in the most deprived groups and from minority ethnic backgrounds are least likely to attend cervical screening,^9^,^11^ and evidence shows that cervical cancer incidence in England is 65% higher in the most deprived quintile than in the least deprived, based on the income domain scores of the Indices of Multiple Deprivation datasets.^12^

A study showed that 64%–65% of women who migrated to the UK as children or adults were cervical screening non-attenders compared with 30% non-attendance for women born in the UK.^9^ Documented barriers to accessing screening include fears around discomfort of the test, embarrassment and desire for a female healthcare practitioner,^13 14^ and though these common factors have also been highlighted in the migrant population, little work has been done to understand the specific challenges of women from minority backgrounds including migrant women of Muslim faith.^15 16^ These patients may face additional concerns about language barriers, perception of the UK healthcare system and cultural norms and values.^17 18^

Self-sampling for hrHPV screening has been shown to be up to 95% acceptable in the UK^14 19^ and up to 97% globally^20 21^ and may double the likelihood of engagement with the NHSCSP.^22^ A focused literature review found self-sampling to be highly acceptable in minority migrant women globally^23^ but highlighted the need for further research on migrant populations and development of recommendations to further mitigate barriers to self-sampling and cervical screening.

The results of the YouScreen feasibility trial showed a 22% increase in non-attenders screened per month.^24^ This may reduce the need to attend a healthcare facility to have the sample taken, give more privacy and reduce embarrassment and pain associated with a clinician-taken sample, which requires a speculum examination.^20 21^ It is also likely to reduce the burden on primary care by reducing the need for face-to-face appointments and may reduce the cost of the NHSCSP overall.^25^ Despite well-documented research into the factors that may be barriers and facilitators to engagement with the NHSCSP,^9 13 17^ there has been limited exploration of how acceptable novel self-sampling strategies may be to specific groups within the diverse population of the UK. Novel strategies of cervical screening need to be carefully explored to ensure that they are acceptable to patients across different ethnic backgrounds, faiths and communities, to provide equitable access to the NHSCSP and therefore reach those least likely to attend.

St George’s University Hospital NHS Foundation Trust delivers care for patients from a wide catchment area including South London and Surrey, with an ethnically diverse population: 59% and 52% non-white British ethnicity according to 2021 UK Census data (for Merton and Wandsworth London boroughs respectively).^26^ Therefore, this study aimed to build on the existing international data on the acceptability of self-sampling for cervical cancer screening specifically within the migrant Muslim population in the UK, which has not been previously explored. We have defined migrant women within this study to mean international migrants, born outside of and residing in the UK, which may include refugees, asylum seekers and family migrants.^27^ Using robust qualitative research methods, we aim to place underrepresented communities at the heart of the research process, understand the specific challenges facing this population and develop strategies to improve access to cervical cancer screening.

## Methods

### Study design

We conducted qualitative in-depth semi-structured interviews via audio recorded MS Teams video call with written informed consent, with the option to have camera turned off. Interviews were carried out between 9 February 2022 and 27 April 2023.

We acknowledged that post COVID-19 pandemic, some vulnerable groups still preferred to limit face-to-face meetings, though including the option of face-to-face meeting if requested, and of focus group discussion if preferred, but none of the participants preferred the focus group option. Translation services were available on request via The Language Shop.

The primary aim was to explore the acceptability of low vaginal hrHPV self-sampling for cervical cancer screening among migrant Muslim women in Southwest London compared with traditional healthcare worker-acquired samples. The secondary objectives were to explore participants’ knowledge and awareness of cervical cancer, perceived barriers to cervical cancer screening access and motivators for engagement with the cervical cancer screening programme. The final aim was to explore the knowledge, concerns and acceptability of HPV vaccination, for which the results will be reported separately.

Participants were remunerated for their time associated with attending the interview, with a £25 shopping voucher for all participants who completed the interview, in line with National Institute for Health and Care Research guidance.^28^

### Study population

*Target population*: Women and people with a cervix in the Southwest London region. Participants were not excluded based on time residing in the UK or ability to speak/read English.

Inclusion criteria:

- Women and people with a cervix aged 25–64 years (screening age in England)
- Born outside the UK
- Currently residing in the UK
- Muslim faith
- Must be able and willing to give informed consent

Exclusion criteria:

- Suffered from cervical cancer
- Temporarily in the UK for holiday, visiting friends/family or other reasons.
- Individuals who may lack the capacity to consent, as determined by the mental capacity act framework.

### Participant recruitment and interview design

Participants were recruited through community engagement, whereby initial relevant organisations aimed at providing advice to migrant and Muslim women were identified with our collaborators from the City St. George’s University of London Migrant Health Research Group. These organisations were then contacted by email, inviting them to collaborate on the project and help with recruitment.

The topic guide (online supplemental information 1) and interviews had four main sections: knowledge on cervical cancer, knowledge and views on cervical cancer screening, knowledge and views on self-sampling and knowledge and views on HPV vaccination. The data collected on HPV vaccination were analysed separately for this paper.

The topic guide was developed initially based on the ‘Cervical Cancer Awareness Measure (Cervical CAM) Toolkit’ developed by the University College London Health Behaviour Research Centre, in collaboration with the Department of Health Cancer Team and the Eve Appeal, with funding from the Eve Appeal. It forms part of the Cervical Cancer Awareness and Symptoms Initiative.^29^ It is based on a generic CAM developed by Cancer Research UK, University College London, King’s College London and Oxford University, in 2007–2008. A basic outline of the topic guide was proposed and then developed within the research team and tailored to include questions on self-sampling and vaccination and to make it more suitable for interview-style questioning. It was then further reviewed by community leaders within the target population as discussed in the ‘Patient and Public involvement’ subsection below.

Purposive and snowball sampling techniques were then employed, where community leaders identified the initial potential participants and approached them to gain verbal consent to pass on their contact details to one of the study investigators. We then approached the participants to discuss the study further (translators available on request) and organise a provisional date for an MS Teams interview, if they agreed to go ahead after reading the Participant Information Sheet. Participants could return their written Informed Consent Form by email or had the option of a separate recorded verbal consent, in case of difficulties with technology or literacy.

At the time of the inception of the study, COVID-19 transmission and shielding were still of some concern and provided the basis for remote interviews. By the commencement of data collection, the COVID-19 restrictions had been fully relaxed, so the participants were also offered face-to-face individual interviews or focus groups. However, some individuals and those shielding would still prefer to limit in-person meetings.

When community snowball sampling slowed, a second round of recruitment was initiated via an article in the St. George’s Hospital Charity newsletter and on the Muslim Women’s Network Hub UK website,^30^ which sparked a second wave of participants with further snowball sampling.

### Data collection and analysis

In-depth semi-structured interviews (by SW and NMA), using the topic guide as a basis, were carried out lasting 45–60 min, via MS Teams video call with automatic transcription service. The interviews were carried out in the English language, but with access to professional interpreters who could join the MS Teams call. Transcripts were then checked against the recording for accuracy by the interviewer and anonymised. Data collection ended when thematic saturation^31^ was reached, and no novel codes or themes were arising within the analysis. The first round of 13 interviews was actually analysed as below, as it was felt thematic saturation had likely occurred, but the small sample size was highlighted during discussions, so five further interviews were carried out and analysed after each one, with no new themes or relationships occurring, confirming saturation.

Data were then analysed using framework analysis with an inductive approach^32^ in NVivo 14, with familiarisation, coding and theme identification, allowing matrix development^33^ by the primary researcher (SW), which was then summarised in Microsoft Excel. Preliminary themes were discussed and reflected on (by three reviewers SW, NMA and YA) after the first phase of recruitment, and any discrepancies in coding and themes were discussed and resolved. The matrix was then improved and applied to further transcripts with any new themes added to the matrix, after which findings were presented to and assessed by a further reviewer (SH) before a second phase of analysis where final themes were refined.

With regard to researcher characteristics, SW is a gynaecologist with an interest in increasing access to cervical screening, and NMA is a researcher with an interest in developing novel methods for cervical screening and has worked with under-represented women in Malaysia. Both researchers who undertook the interviews understand the CSP and have an interest in increasing access to screening in communities facing health inequalities. This was not felt to affect the results of transferability. Co-authors NMA, YA, HS, KH and SK have experience in point-of-care diagnostics and cervical screening as well as colposcopy and have experience in working with hard-to-reach groups of women in Malaysia. Co-authors SEH, AD, AC and SH are part of a Migrant Health Team within City St. George’s, who have extensive experience in qualitative work, particularly around vaccination uptake in under-represented communities, including HPV, and this collaboration provided important insight into the rigour and format of qualitative research and the important nuances and considerations for working with migrant women.

We used the SRQR reporting guideline^34^ to draft this manuscript and the SRQR reporting checklist when editing, included in online supplemental information 2 (S2).

### Patient and public involvement

To ensure that public and patient involvement and engagement is central to the study, a focus group was carried out with four community leaders from Mushkil Aasaan Tooting, Wandsworth Community Empowerment Network, Mindworks UK and the St. George’s Hospital Chaplaincy and Spiritual Care team. The project aims and strategies for recruitment were discussed, and the proposed interview topic guide (online supplemental information S1) was discussed and circulated, and feedback collected to ensure that it was sensitive to our target population and relevant to the study.

## Results

Twenty participants were approached and consented to the study, though two did not attend the interviews and did not respond to a reminder and, hence, were not further contacted and withdrawn from the study. We carried out 18 interviews. Thirteen of these interviews were carried out between February and August 2022, and five were carried out between March and April 2023.

Three of the interviews were carried out with Arabic interpreters assisting with translation, the remaining interviews were carried out in English, as the participants felt proficient even if not their first language.

### Demographic data

We collected demographic data to allow better understanding of the background, culture and beliefs of the participants and allow comparison with other studies into self-sampling acceptability with different and potentially less under-represented groups of people, as outlined in table 1.

**Table 1:** Summary of participant demographics

Characteristic	N
Age (years)	
25–34	7
35–44	4
45–54	6
55–59	1
Place of birth	
South Asia	8
North and East Africa	7
Middle East	3
Marital status	
Single	3
Married	11
Divorced or separated	4
Employment	
Asylum seeker/unemployed	3
Homemaker	6
Part time	4
Full time	5
Years living in the UK	
0–10	5
11+	13

### Analysis

#### Barriers and motivators to cervical screening

Three of the participants had not undergone cervical screening performed in the 5 years before the interview (but had previous screenings). Of those who had undertaken screening, most were performed by a nurse at the general practice (GP) clinic, with two being performed by a gynaecologist.

Barriers and motivators were then explored in depth and themes developed within the matrix. Several overlapping themes emerged, organised into three main strands: knowledge and education, family and community support, and health attitudes. These themes demonstrate how personal, cultural and organisational factors interact to shape women’s engagement with cervical screening.

### Theme 1: Knowledge and education

Across the participants, there was a lack of knowledge of eligibility for the cervical screening programme, intervals between screens and what HPV was and why the UK screening programme now primarily looks for its presence as a triaging tool. Few participants recognised that cervical cancer could be asymptomatic, and many expressed low confidence in recognising warning signs. Misinformation and lack of access to trusted, evidence-based sources further reinforced uncertainty:

‘*If you get pains, don’t think it’s nothing; it can happen to anyone. You just don’t have to be married to get cervix cancer’ *(Participant A, 25–34 years)*.*

This quote demonstrates the prior false belief that one would need to have had penetrative sex (ie, be married in Muslim faith) to be exposed to HPV and be at risk of HPV exposure.

Importantly for this group of migrant women, they felt that not speaking English proficiently and limited familiarity with the UK healthcare system amplified these challenges. The most recently arrived participants described being unaware of how to access primary care or ask for help, identifying the first months post-migration as a particularly vulnerable period when culturally sensitive education was lacking.

Conversely, participants felt that access to clear, evidence-based health information and education, in their own language, by a trusted member of their community or a trusted healthcare worker was strong motivating factors:

‘*I think it’s just a matter of converting that information in a different language and making it accessible. But I think people would be on board with it as soon as they have that kind of information put in front of them’* (Participant A, 25–34 years*).*

They also acknowledged the positive effect that media campaigns and knowledge of high-profile cervical cancer sufferers (eg, Jade Goody^35^) was likely to have on their engagement and interest in the screening programme.

Therefore, health literacy and linguistic accessibility of educational sources emerged as pivotal factors that could either hinder or facilitate screening engagement.

### Theme 2: Family and community support

Cultural and religious norms strongly influenced women with regard to decision making around screening. Having an intimate examination, the association with sexual activity, particularly for unmarried women and feeling unable to talk about it with friends, family and community leaders often created tension. Family and community influences either prevented or wholly empowered the participants to discuss screening and worrying symptoms, and even in more open households, marital status remained a gatekeeper to perceived appropriateness of screening

‘*The cervix and all of this stuff… It was more open conversation in our home. …when my mom and my two sisters were girls, so we always talked about it. … And I always tell (my sister) about it and say, listen, just because you can’t do it now because you still haven’t married, (doesn’t) mean that you shouldn’t’* (Participant B, 25–34 years*)*.

A number of participants also discussed that receiving their screening results if they were still living at home was problematic as a result of being unsupported by their families. The results are posted to their home address, and they found it difficult to request the results via a different, more confidential method.

Another significant barrier was the sex of the healthcare professional undertaking the screening because of cultural expectations, and uncertainty around this might prevent attendance, as their community may not support them or see the examination as unnecessary. Similarly, having family members who worked in healthcare was often empowering, as it provided access to reliable information and positive role modelling—linking back in with the theme of access to knowledge and information.

Gender inequality and the feeling that it was a ‘female problem’ because of feeling unsupported by their partners and husbands within their family setting, as well as males in their extended community, were also felt to be a barrier to accessing screening:

‘*I always walk out angry when I have to go for something related to my vagina, … I complain to my husband as well, saying, you know, I just find it so crazy that we have to go through these painful procedures to get contraception fitted or, you know, take ownership of our body’* (Participant A, 25–34 years*).*

Overall, family and community norms acted as both barriers and facilitators—capable of either silencing discussion or fostering empowerment through open dialogue and supportive relationships.

### Theme 3: Health attitudes

Participants identified cultural attitudes toward health and illness as key influences on screening behaviour. Fear and denial of ill health and disbelief of cancer were common, or feeling ‘well’ and not prioritising time for a screening appointment:

‘*Community majority is just a fear of having (it) … we don’t believe in it because when it comes to cancer in our community, they don’t tend to believe it’* (Participant C, 25–34 years*).*

This fatalistic outlook was compounded by fear of pain associated with an intimate examination, embarrassment, taboo and shame of being exposed. Negative experiences of screening, described as painful or demeaning, intensified avoidance:

‘*It was far from gentle for me, and I did say to her I’ve had a coil fitted before and I was screaming and crying. … but when you go in there and you walk into there before you go to the room before you sit in that (chair), you feel embarrassed, and you feel uncomfortable’* (Participant A, 25–34 years*)*.

Trust in healthcare professionals was therefore critical. Participants who had developed long-term, trusting relationships with their GP or nurse expressed greater confidence in attending screening, underscoring the importance of continuity of care. Individual agency or feeling of being responsible for their own health to be present for their children and set a good example for their children was also a strong motivating factor:

‘*It’s safer to keep health checking yourself because what you’re doing, you’re doing it for your future great-grandchildren, really’* (Participant B, 25–34 years*)*.

COVID-19 also influenced health attitudes at the time of the interviews, and several participants were concerned about attending a public place for screening and putting themselves at risk of exposure to COVID-19. Several appointments had been delayed during the pandemic because of the pause in screening and concerns about the impact that this might have.

#### Views on low vaginal self-sampling

Only 4 of the 18 participants had heard of self-sampling for cervical screening before the interviews, and most required an explanation. Once explained, 8 of the 18 participants (44%) felt they would rather take a self-sample than attend for HCW-taken cervical screening samples. Interestingly, all the women felt that self-sampling had some benefits and was likely to be beneficial for some women, even if they preferred a traditional screening.

Five subthemes emerged: preference over intimate examination, technique, convenience, receiving results and influence of COVID-19.

### Theme 1: Preferential to an intimate examination

The participants overwhelmingly felt that self-sampling was likely to be less invasive, less painful and less embarrassing than a speculum examination. Some voiced concern over missing other pathology than might be seen during an examination or a chance for a broader health check lost.

*‘I don’t feel comfortable someone (doing) it for me, to examine me, so to do it myself, I’m gonna feel more comfortable.’* (Participant D, 25–34 years)

### Theme 2: Technique

There was a concern over limited awareness of their anatomy, compounded by the cultural taboo of touching one’s genitals, and this would hinder their ability to perform the test correctly and affect the accuracy of results:

‘*You’re not supposed to look at it. It is improper, so how would that work in terms of taking your own swab? Would that prevent them from doing a proper job?’* (Participant E, 25–34 years)*.*

Conversely, there was an emphasis that they would feel much more confident and would consider self-sampling as their primary form of screening if there were clear instructions with a diagram or video. They also felt if they could be directly supervised taking the sample for the first time by a HCW to ensure correct technique and that this would legitimise the process.

### Theme 3: Convenience

The ability to perform the test in the comfort of their home was found to be a positive factor, particularly for women managing childcare or employment demands. Even those who preferred an HCW-taken sample acknowledged that this was a major advantage of self-sampling.

However, some were worried that self-sampling might reduce motivation, leading to procrastination or missed return of samples. There was also concern about postal reliability:

*‘There will be a bit (of) laziness to do it… For you to do it and then post it as you are mum, you’re running. It’s just like timing is… It would be longer to be done.’* (Participant F, 55–59 years)

### Theme 4: Receiving results

The importance of being able to confidentially receive the results of self-sampled screening was critical. It was felt that text message results were highly convenient and preferential to postal results. It was noted, however, that if there was an abnormal result, a simple text message might cause alarm and that a face-to-face or telephone appointment was preferential in these circumstances to provide time for reassurance and explanation.

’*If it’s OK, text message, everything’s fine. But if it’s not OK, I would prefer to get a phone call… You know, you’re gonna start panicking, … I would prefer to have that phone call for someone to … kind of go through everything with me.’* (Participant G, 25–34 years)

### Theme 5: COVID-19

The pandemic had a paradoxical effect. Although it disrupted routine screening, it also normalised home-based testing and self-management of health. This was felt to have a positive influence on the participant’s ability and confidence to perform other tests themselves as a result of taking their COVID-19 swabs, which could positively influence acceptance of self-sampling.

*’It’s quite nice that you know, [it’s] just the way we do the [COVID-19] PCR test.’* (Participant D, 25-34 years)

## Discussion

This study is the first of its kind to gather the views of migrant Muslim women in the UK on hrHPV self-sampling for cervical screening. This group of women is particularly at risk of healthcare inequity given the additional challenges of having to understand a new healthcare system along with language barriers and cultural differences.^9 17 18^

Limited work has been carried out in the UK to evaluate acceptability and practicalities of the use of hrHPV self-sampling devices in minority groups, particularly in Muslim women—a small focus group study with 28 Muslim women was performed and noted generally positive attitudes towards cervical screening, but with most women preferring to see a clinician than undertake a self-sample.^18^ However, a limitation of focus group studies is that not all the women can express their views, and the results may reflect that of a smaller number of more vocal participants. Therefore, this study is significant in that each participant was interviewed individually, with the element of anonymity, to empower them to express their views without fear of judgement from their religious leaders and community.

There was a wide range of ethnicities, ages, languages spoken and employment and family status among the participants, despite being from a specific community within southwest London. This represents a diverse range of cultural influences and drivers and therefore captures as many themes as possible within theoretical saturation. This gives an invaluable insight into how to increase engagement with cervical screening and the acceptability of a possible introduction of hrHPV self-sampling into the screening programme.

The unifying theme here is information giving and education; strategies to engage migrant Muslim women with cervical screening, no matter the method, are likely to lie in access to trusted information sources such as community champions who speak the language of the women that they work with, understand the cultural nuances and have access to resources that women can trust and understand. Then, women who engage with the screening programme but do not wish to have an intimate examination are more likely to trust and feel confident enough to perform self-sampling and know that they can receive the results in a confidential manner.

This is consistent with previous work on understanding barriers to screening in ethnic minority women. A lack of knowledge and understanding and misinformation within their community were common barriers, compounded by being unable to access information in their language.^9 13 17 18^ Similarly, concern over the association of cervical cancer and HPV with sexual activity outside of marriage and the taboo of an intimate examination, a desire for a female health practitioner and feeling unsupported by their partners, family or community were also consistent barriers to screening in this study.^9 13^

Cultural norms and views on ill health as fate or destiny and distrust of healthcare workers were noted in this study, and the issue of younger women living with their parents receiving invitation letters and results to their family home and the associated lack of confidentiality were key novel barriers to consider when trying to understand reasons for non-attendance. This raises the importance of confidentiality and allows women alternative methods to not only undertake screening but also receive their results and ‘opt-out’ of results coming to their home address.

Conversely, access to clear evidence-based information, in their language, by a trusted member of their community or a trusted healthcare worker was strong motivating factors, supported by work in previous studies of migrant women in the UK and internationally,^1736^,^38^ as well as advance knowledge of access to a female healthcare worker, or a strong trusting relationship with their GP or primary and secondary care team. Women’s agency over their health was also key, with role modelling from their community or from family members who worked in healthcare a strong motivational tool.

With regard to the acceptability of low vaginal self-sampling for cervical screening, migrant Muslim women felt that although it was clearly beneficial for some women as it was likely to be less painful, less embarrassing and more convenient than a HCW-taken sample, only 44% stated that they would prefer to perform a self-sample. The key concerns were incorrect technique and inaccurate results because of limited awareness of their anatomy, cultural taboo about touching an intimate area and being less motivated to perform the screening without an appointment. These barriers to self-sampling were also reported in two recent systematic reviews^21 39^ and tie back in with the importance of education on technique and anatomy before self-sampling to instil confidence in the user. Being lost to follow-up in the event of an abnormal result because of fear of the implications without a face-to-face explanation was also described as an important barrier.

The COVID-19 pandemic interestingly had the effect that despite delays in screening appointments, women felt they had some experience of taking control of their health and specifically in taking their own swabs and following enclosed instructions, which made them feel *more* empowered to explore self-sampling for screening.

These findings re-iterate the importance of careful consideration of the way in which self-sampling is offered having been included in the cervical screening programme in the UK, as while minority ethnic and migrant women face more health inequality, self-sampling may be optimally offered to non-attenders presenting to primary care as suggested by Lim & Sasieni, 2015,^40^ the YouScreen trial,^24^ where a clear explanation can be given by a trusted health professional. To allay fears of incorrect technique and inadequate results, discuss the way in which the results can be received and the reasons the screening is required. This study reaffirms the need for more work to be undertaken in the community to improve education on HPV and cervical cancer and help women in understanding the importance of screening before marriage as well as in long-term relationships, and even after previous normal screening, to ensure that this group of women is not lost to follow-up because of misinformation.^9^

## Limitations

This was a relatively small study of 18 participants. Although idea saturation with the in-depth interviews was reached, most of the women had attended screening in the last 5 years, and all had attended screening at some point. This may be under-representative of the views of a potential target population of screening non-attenders for low vaginal self-sampling, though still represents an under-screened community of women.

Only three of the participants were unmarried with no children; given the strong influence of social norms and stigma around attending cervical screening before marriage and HPV vaccination in school-age children, because of its association with sexual activity, this subgroup is likely to require much larger numbers for exploration and more in-depth future research. Given the increased privacy and reduced perceived shame and embarrassment associated with hrHPV self-sampling, a solution for the particularly intimate nature of cervical screening may be finally overcome.

Additionally, only low vaginal swab self-sampling methodology was discussed, and urine self-sampling may also provide an acceptable and non-invasive form of cervical cancer screening.^41 42^

## Conclusions

Low vaginal self-sampling has been found to be a highly acceptable mode of screening for hrHPV among the under-screened Muslim migrant population.

The recent UK NSC recommendation supporting self-sampling for cervical screening non-attenders offers an unprecedented chance to close screening inequalities; however, it brings logistical and ethical challenges. Ensuring equity will require that NHS England and local authorities deploy self-sampling with targeted strategies, prioritising linguistically appropriate materials, partnership with community leaders and flexible access points (at-home, primary care and community settings).

To optimise hrHPV vaccine uptake, it is essential to bridge gaps between policy and lived experience.

Interventions should prioritise the following:

- Co-design of education materials in collaboration with migrant communities.
- Provision of translated resources and professional interpreters in all screening encounters.
- Ensuring confidential modes of result delivery and alternative follow-up pathways for women concerned about privacy in the family home. National digital campaigns may play a supporting role, but durable change is most likely if efforts are embedded in local, face-to-face advocacy and healthcare partnerships.

## Supplementary material

10.1136/bmjph-2025-003254 online supplemental file 1

10.1136/bmjph-2025-003254 online supplemental file 2

## Data Availability

Data are available in a public, open access repository.
