# Supplementary material for: Understanding knowledge, beliefs, values and barriers towards cervical cancer screening and self-sampling amongst migrant Muslim women in Southwest London: an in-depth qualitative interview study
Source: BMJ Public Health. 2026 Jan 9;4(1):e003254. doi: 10.1136/bmjph-2025-003254 (PMC12815087; doi:10.1136/bmjph-2025-003254)
Supplement: online supplemental file 1 [file bmjph-4-1-s001.docx]

**Topic Guide Semi-Structured Interviews: Version 4.0**

**Understanding knowledge, beliefs, values and barriers towards cervical cancer screening, self-sampling and HPV vaccination amongst migrant Muslims and stakeholders in South West London: An in-depth qualitative interview study**

1. Introduction

*Welcome*

- Thank you very much for agreeing to participate in this study, and for sharing your views and experiences of cervical screening, self-sampling and HPV vaccination.
- I’m a researcher at St George’s, University of London
- Usually this will take around 45 minutes

*This study*

- We are asking you to share your experiences so that we can understand the ideas, concerns and preferences among women in our local population around cervical screening, cervical cancer, and the HPV vaccination programme.
- We are focusing on women in the Muslim community who are not born in the UK, because we think it is important to try to reach people in your community so that we can improve uptake of cervical screening and empower women to participate in screening in the future, by identifying potential barriers.
- We are also trying to find out your views on how acceptable certain new interventions would be, to see if we could help to overcome these barriers in the future.
- Just to be clear, we won’t be offering you any treatment today, and we are not part of your medical team from St. George’s Hospital.
- Everything you say will be totally anonymous and is part of a wider series of interviews. If at any point you would like to take a break or stop entirely, that completely fine. Also if you don’t feel comfortable with certain questions, we can skip them.
- If you want to rearrange for another day or withdraw from the study altogether that’s also completely fine and it won’t have any impact on the care you receive.
- We’d like to understand your experiences, how you felt and if there are things we can do to improve your experience. It’s important that you know there are no right or wrong answers to any of the questions I’m going to ask you today.

*Questions*

- Do you have any questions for me before we start?

*Introduction*

- I’m going to ask you some questions about your background, your knowledge on cervical cancer, cervical cancer screening, and HPV vaccination. I am also going to ask you about your own experience of cervical cancer screening, if you have had any.

1. Introductory questions

To start with I have some quick questions about yourself:

1. What is your age?
2. Where were you born?
3. How would you describe your ethnicity? (Possible prompts: Asian or Asian British (within this Indian, Pakistani, Bangladeshi, Chinese, Any other Asian background), Black, Black British, Carribean or African, Arab, Multiple Ethnic groups (White and black African, White and Black Carribbean, White and Asian)).
4. How long have you been living in the UK?
5. What is the main language spoken at home?
6. What is your family status? (Married, single, any children?)
7. What is the highest education qualification you have obtained?
8. Are you currently:
   - Employed full-time
   - Employed part time
   - Unemployed
   - Self-employed
   - Full-time homemaker
   - Retired
   - Still studying
   - Differently abled
   - Not able to work due to a health condition
   - Prefer not to say
9. Have you, your family or close friends had cancer?
10. Do you have a permanent and full registration with a GP in the UK?
11. Core questions

***Knowledge on Cervical cancer (based on Cervical Cancer Awareness)*** [*https://www.cancerresearchuk.org/sites/default/files/health_professional_cervical_cancer_awareness_measure_toolkit_version_2.1_09.02.11.pdf*](about:blank)*)*

1. Have you heard of cervical cancer? (Prompt: May need to explain that the cervix is the neck of the womb and has to open during birth to allow the baby to be born.)
2. Are you familiar with any warning signs and symptoms of cervical cancer?
   - Prompt: If participant unable to respond, read out the symptoms;

Vaginal bleeding in between periods, persistent lower back, persistent vaginal discharge, discomfort or pain during sex, menstrual period that is heavier than usual, vaginal bleeding after menopause , vaginal bleeding during or after sex, unexplained weight loss blood in stool or urine, persistent diarrhoea. Participant answer yes, no or unsure.

1. Is cervical cancer screening something that you can talk about with other women in your family or female friends? Would you recommend it them/encourage them to participate in cervical cancer screening?
2. Are you aware of any particular age group which might be more likely to develop cervical cancer in the UK?
   - Prompt: Can it affect girls, younger women or older women?
3. If you had a symptom that you thought might be a sign of cervical cancer how soon would you contact your doctor to make an appointment to discuss it?
   - What might stop you from contacting them?
4. Can you think of any things that might make a woman more or less likely to develop cervical cancer?
5. Do you feel confident that you would notice a cervical cancer symptom? What makes you unsure?

**Cervical Screening** *(Knowledge, beliefs, values and barriers)*

- 1. Have you heard of the NHS cervical cancer screening programme or ‘Smear Test’?
  2. If YES have heard of ‘Smear Test’– do you know what the Smear Test is for?
  3. If yes, are you aware of what age you are usually first invited for cervical screening in England?
  4. Have you had a cervical smear or HPV DNA screening for cervical cancer in the last 5 years?
- Yes, get further details e.g. where was it done and proceed to **Question 5**
- No, please proceed **to Question 7**
  1. Overall, how was your experience of the cervical Pap smear?
- Prompt: How do you feel when you had the procedure? E.g. pain, discomfort, embarrassed
  1. Who performed the Pap smear? How was your experience? Proceed to **Question 7**
- Prompt: Did they explain the procedure to you? Were you confident that the Pap smear was done correctly? Did you have any questions that you felt you could not ask the healthcare professionals?
  1. (IF NO SMEAR LAST 5 YRS) Could you tell me a bit about why?
- Prompt: Did you get an invitation letter from the NHS? Was there something that you are unsure about the test? Some reasons- did not get any invitation, not confident/fear, no time to attend the appointment, no symptoms therefore I thought the test was not necessary, not appropriate in my culture, lack of knowledge or information on the topic, I did not know it was available to me.
  1. Have you ever heard of HPV (Human Papillomavirus)?
- Yes 🡪 Are you aware of HPV screening is now part of the Smear Test?
- No – Explain to the patient; HPV is a common virus that affects over 80% of adults in their lifetime. In most cases a person’s immune system naturally clears HPV with no long lasting effects. However in a small proportion, persistent HPV infection can result in persistent inflammation that can lead to cervical cancer. We now test for high risk types of HPV on the sample taken during your ‘Smear Test’.
  1. Have you heard of HPV **self-sampling** for cervical cancer screening?
- Yes
- No – **Explain to the patient**; HPV self -sampling method used to screen for cervical cancer. It allows women to take their own sample for cervical screening using a vaginal swab (like a long cotton bud).
  1. How would you feel about collecting your own vaginal swab for HPV testing?
- Prompt: Would you be comfortable doing it yourself? Do you think it would be easy and convenient to perform? Would you feel embarrassed? How confident are you that you will be able to collect this sample correctly?
  1. Are there any reasons why you or someone you know has declined or not attended cervical cancer screening in the past?

Prompt:

- Fear the test may be uncomfortable/painful
- Worried about embarrassment having to be examined by a healthcare worker
- Worried about being examined or treated by a male doctor
- Worried about being diagnosed with cancer/pre-cancer that might require treatment
- Worried that my partner/family may disapprove
- Worried about the cost of travelling to hospital for the test and follow up if I needed treatment
- Worried that the test or treatment if needed could affect fertility
- Worried about taking time off work
  1. In theory, would you be willing to do the HPV self-sampling test if it was offered to you and you received appropriate guidance and support to perform the test? Just to be clear – we are not currently offering this type of test, but may do in the future.
- Why?
- Would it make a difference if you did it at home, in the community (e.g. a Women’s only health clinic), or in a clinical environment?
  1. Would you prefer to do a self-swab for HPV testing, or attend for a healthcare worker-taken swab?
     - Why?
  2. How would you feel about receiving a test by post with an information leaflet/video link, performing the test at home and then posting it back to the hospital lab for testing? (a bit like a COVID-19 self-test)
  3. How would you feel about receiving your results (if normal) by a text message/app (similar to the NHS app for COVID-19) provided you had a helpline to call if you had further questions?

***HPV Vaccination*** *(Knowledge, attitudes, beliefs, values and barriers)*

1. Have you heard of of the HPV vaccination? Where did you hear about the HPV vaccine?
   - Prompt: What can you tell me about the HPV vaccination programme? Do you know what age groups are offered HPV vaccination?
2. Do you know what the HPV vaccine does?
   - Prompt: Are you aware of how effective the vaccine is against cervical cancer? Do girls who have had the HPV vaccine still need to have regular cervical cancer screening tests?
3. Have you or your child received the HPV vaccine? If yes, continue. If no, please proceed to **Question 4**
   - Prompt: What helped you (or your children) decide to get vaccinated? Did your GP/ HCW recommend the HPV vaccines to you (and your children)? Was the process easy?
   - Prompt: Did they explain the vaccine to you? Was there anything about the explanation that you did not understand?
4. IF HAVE NOT HAD VACCINE: Have you or your child ever been offered the HPV vaccine?
   - What would you say are the key barriers to vaccination for you? And others around you? [Prompt: language, culture, time, religion, knowing where to go?]
   - If No: Do you think you would take the vaccine if it were offered, and why?
5. If you wanted a vaccine for yourself would you know where to go, if so, where/how would you go about it?
6. In what way does the UK vaccination system differ from your home country?
7. Are you aware of problems with vaccine uptake in your communities, any particular groups?
8. *Have you ever avoided accessing vaccination on the NHS because of fear of cost/being told you weren’t entitled/ fear of discrimination? [try to not to make too leading where possible]*
9. Do you feel you have enough awareness of the HPV vaccinations, do you have access to any educational material? Do you think this is needed?
10. How do you think we could engage people who have recently arrived in the UK to have HPV vaccines/improve access?

*Overall*

[If there is time and the participant would like to, repeat some of the above questions for other significant events and periods before/during/after migration]

1. End

- Is there anything else you want to add/think I should have asked?
- Do you have any questions for me?
- Would you like me to send you a copy of the final report once it has been completed?
- Thank you so much for taking part, I really appreciate your time.
